# Effects of biguanides on oxidative phosphorylation

**DOI:** 10.1186/2049-3002-2-S1-O28

**Published:** 2014-05-28

**Authors:** Judy Hirst

**Affiliations:** 1 MRC Mitochondrial Biology Unit, Cambridge, UK

## 

Metformin, and the related biguanides buformin and phenformin, have been proposed to act by inhibiting respiratory complex I, causing the cellular energetic stress that activates AMP-kinase and initiates a multitude of cell-lineage-specific effects, including inhibition of gluconeogenesis in hepatocytes. However, the molecular mechanism of biguanide action on complex I is not known, limiting understanding of pharmacological effects on this proposed primary target. Biguanides have also been proposed to affect reactive oxygen species production in cells, a respiratory-chain linked effect - but there is no consensus even on whether they increase or decrease it. In this talk I will discuss the results of experiments on a hierarchy of systems, from purified respiratory enzymes and membrane preparations, to mitochondria and cells, to define the functional effects of biguanides on the oxidative phosphorylation system. The aim is to define and link the molecular effects of biguanides on specific enzymes to their known effects on cellular and signalling pathways.

